# Spatially limited pathogen pollution in an invasive tick and host system

**DOI:** 10.1007/s10530-024-03291-9

**Published:** 2024-04-23

**Authors:** Carrie E. De Jesus, Madison E. A. Harman, Amber Sutton, Stephen Bredin, Christina M. Romagosa, Samantha M. Wisely

**Affiliations:** 1 Department of Wildlife Ecology and Conservation, University of Florida, Gainesville, FL USA; 2 Biology Department, George Mason University, Fairfax, VA USA

**Keywords:** *Amblyomma rotundatum*, Cane toad, Core-periphery distribution, Enemy release hypothesis, Founder effects, Invasive species, *Rickettsia bellii*, *Rhinella marina*, *Rhinella horribilis*

## Abstract

Expansion of global commerce has facilitated pathogen pollution via the transportation and translocation of invasive species and their associated parasites and pathogens. In Florida, imported cane toads (*Rhinella horribilis*) were accidentally and intentionally released on multiple occasions. Early populations were found to be infested with the invasive tick, *Amblyomma rotundatum*, yet it is unknown if these ticks dispersed with their hosts as cane toads spread throughout much of the state. The objectives of our investigation were to (1) determine if there are fewer tick infestations on toads at the periphery than at the core of their distribution as predicted by founder effect events, and (2) identify if ticks were infected with exotic pathogens. We captured toads from 10 populations across Florida. We collected ticks, vent tissue, and tick attachment site tissue from each toad, then tested samples for bacteria in the genus, *Rickettsia*. We found that 3/10 populations had toads that were infested with *A. rotundatum*, and infested individuals were in the earliest introduced populations at the core of their distribution. Pathogen testing confirmed *Rickettisa bellii* in ticks, but not in toad tissues. Haplotype networks could not clearly distinguish if *R. bellii* in Florida was more closely related to North or South American strains, but host-tick associations suggest that the pathogen was exotic to Florida. Our investigation demonstrated that an invasive species facilitated the introduction of parasites and pathogens into Florida, yet the invasive tick species encountered limitations to dispersal on this host species.

**Supplementary Information:**

The online version contains supplementary material available at 10.1007/s10530-024-03291-9.

## Introduction

The expansion of global trade and travel has created pathways for invasive species and accelerated their spread worldwide (Sakai et al. 2001; Van Kleunen et al. 2015; Capinha et al. 2017). Once introduced, species with traits that support rapid reproduction and dispersal can become successful invaders. Whether intentional or accidental, these introductions can directly or indirectly alter invaded ecosystems resulting in decreased biodiversity, ecosystem function, and ecosystem services (Floerl et al. 2009; Mooney and Cleland 2001).

One way invasive species alter ecosystems is via the co-introduction of non-native parasites and pathogens, i.e. pathogen pollution (Cunningham et al. 2003). An emerging focus of invasion ecology is the study of how host-parasite dynamics change with the introduction of invasive species to a novel area. Parasites and pathogens are ubiquitous in biological communities and can influence both the invasion success of non-native species and the fitness of native species (Prenter et al. 2004). Parasites and pathogens can negatively impact the fecundity of hosts via energy trade-offs that allocate the host’s energy to immune response instead of dispersal, growth, or reproduction (Luong et al. 2017), in turn regulating host population levels (Prenter et al. 2004).

Following the establishment of an invasive host species, parasites and pathogens may be distributed unevenly across the invasive host range due to a variety of ecological processes. Peripheral host populations, those that are on the leading edge of the expansion front, may have lower rates of parasitism due to the stochastic infestation rates of immigrant individuals which may lead to lower rates of parasitism in colonizing populations (Phillips et al. 2010; Barnett et al. 2018). Alternatively, but not mutually exclusively, core host populations, those that established early in the invasion process, may have more parasites than populations along the expanding peripheral edge because habitat at the site of establishment may be of higher quality than habitat in isolated, peripheral populations, leading to higher host and parasite densities at the distribution core. In addition, cross-species transmission of parasites from native or previously established non-native species to the invading species (spill-back) may also facilitate uneven or patchy distribution of parasitism across the landscape (Chalkowski et al. 2018). Thus, patterns of parasite distribution on invasive host species can provide insight into the underlying processes driving host-parasite relationships.

While some parasite populations may struggle to persist during the invasion process, those that successfully become established often share generalist phenotypic traits. Parasites that are host generalists (Ewen et al. 2012) can tolerate variable climatic conditions (Polo et al. 2021) and typically have simple life cycles that require few host species or can reproduce asexually. Many tick species (*Ixodida*) exemplify these traits and are frequently successful invaders (Barré and Uilenberg 2010). Multiple tick species have successfully invaded and established in the United States (Burridge 2011) including *Amblyomma rotundatum,* which originated from Central and South America. *Amblyomma rotundatum* is a 3-host tick species, yet the same host species may support all three tick life stages. *A. rotundatum* reproduces parthenogenically, which streamlines population growth by eliminating the need to search for a mate. *Amblyomma rotundatum* is the one of the most prolific generalist feeders reported in the literature, with 59 reported herpetological host species (De Jesus 2021) and 16 mammal host species (Guglielmone and Nava 2010). This combination of traits has made it a highly successful invasive species.

While it has been hypothesized that *A. rotundatum* was introduced into Florida with its native host, the cane toad (*Rhinella horribilis* formerly *R. marina*, Acevedo et al. 2016, Mittan-Moreau et al. 2022, Oliver et al. 1993), the origins of *A. rotundatum* in Florida are not well understood and are likely complicated. Multiple invasive reptile species that originated from Central and South America have been introduced in the decades after cane toads became established (Krysko et al. 2011; Nava et al. 2017) providing additional potential source populations of *A. rotundatum*, and other non-native reptiles including the Burmese python (*Python bivittatus*) from Southeast Asia and the Peter’s Rock agama (*Agama picticauda*) from Africa (Corn et al. 2014, pers. obs.) have been observed in Florida with *A. rotundatum* infestations*.* Additionally, cases of parasite spillover of *A. rotundatum* have been reported on native snake species like the southern black racer (*Coluber constrictor priapus*) in southernmost Florida and cottonmouth (*Agkistrodon piscivorus conanti*) (Hanson et al. 2007; Corn et al. 2014; Lillywhite and Sheehy 2019) from northwest peninsular Florida suggesting that this invasive tick species is distributed throughout Florida.

**Fig. 1:**
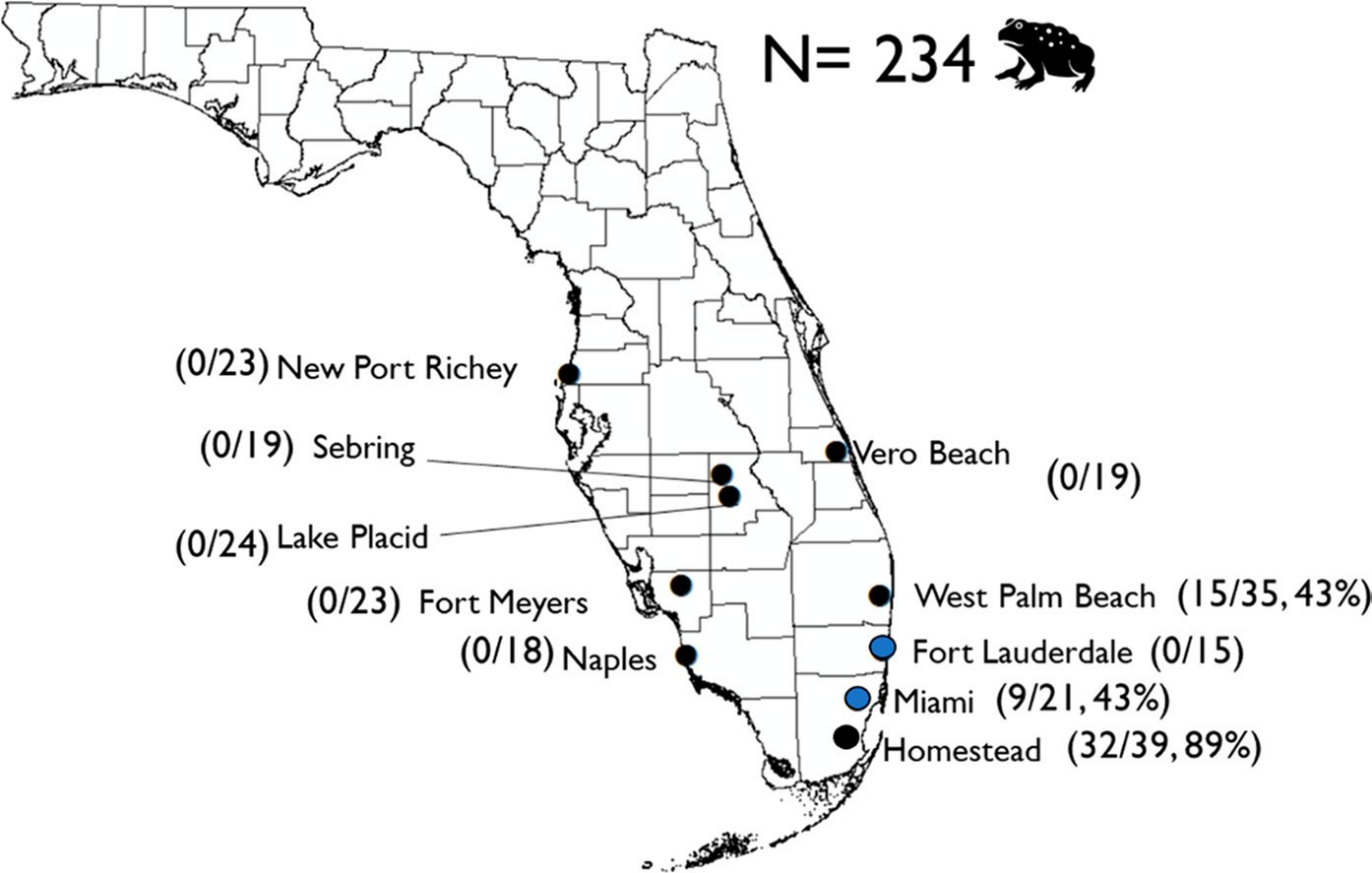
Circles indicate field sites where toads were collected. Blue circles identify sampling sites near introduction locations in Florida. Parentheses display number of individual toads infected with ticks

Cane toads are an infamous invasive species that were introduced into subtropical and tropical climates worldwide in failed biological control attempts for sugar cane beetles (King and Krakauer 1966; Lever 2001). Cane toads were originally introduced into Florida cane sugar fields in Palm Beach County during the 1930s and 1940s, although those populations did not become established (King and Krakauer 1966). Instead, cane toads likely became established in Florida in the 1950s after an accidental mass-release of toads imported to the Miami Airport from Colombia (Krakauer 1968; Oliver et al. 1993). Then in the 1960s, animal dealers intentional released additional cane toads from Colombia in Pembroke Park near Ft. Lauderdale in Broward County and from Suriname in Kendall near Miami in Miami-Dade County (King and Krakauer 1966). Thus, two populations were established approximately 60 km from each other in southeast Florida (Fig. 1). Since these introductions, cane toads have utilized canals and urbanized habitats to establish expanding populations across southern Florida and northwards into central Florida (Meshaka et al. 2006; Wilson 2016). *Amblyomma rotundatum* were first identified infesting invasive cane toads in Miami, Florida in the late 1970’s (Oliver et al. 1993).

Ticks are associated with numerous bacterial pathogens. Bacteria in the genus *Rickettsia* are the most ubiquitous tick-borne pathogen reported globally (Parola et al. 2013; Piotrowski and Rymaszewska 2020). *Rickettsia* bacteria vary in their pathogenicity from causing severe human and animal illnesses to nonpathogenic endosymbionts (Parola et al. 2013; El Karkouri et al. 2022). *Rickettsia* are likely candidates for pathogen pollution because of their unique transmission properties. Unlike some tick-borne pathogens, *Rickettsia* can be transmitted transovarially, i.e., adult female ticks transmit *Rickettsia* bacteria to their eggs and the resulting larvae emerge infected (Horta et al. 2006; Laukaitis and Macaluso 2021). The subsequent life stages can then maintain that infection between molts, referred to as transstadial transmission (Labruna 2009; Parola et al. 2013). Transovarial and transstadial bacterial transmission facilitate transmission from one life stage to another without the tick taking a bloodmeal from an infected host; therefore pathogens can be imported in infected ticks even if those ticks are infesting an uninfected host. All life stages disseminate *Rickettsia* (Parola et al. 2013), although some species of *Rickettsia* require infected hosts to maintain infection in the tick population. Nonetheless, transovarial transmission provides exotic rickettsial species with fewer barriers to overcome during the invasion process than bacteria with other transmission routes.

*Rickettsia* species have been reported in *A*. *rotundatum* within the cane toad’s native range. One commonly reported species is *Rickettsia bellii* (Labruna et al. 2004; Luz et al. 2018; Sánchez-Montes et al. 2019) which has been reported in 19 tick species across the Americas and is considered nonpathogenic (Krawczak et al. 2018). *Rickettsia bellii* infections are maintained by both transovarial and transstadial transmission. This species also has distinct clades for North and South American strains (Krawczak et al. 2018). *Rickettsia bellii* provides a unique model system to observe how *Rickettsia* respond to the biological invasions processes of their tick host.

Cane toads; the tick, *Amblyomma rotundatum*; and the bacterial species, *Rickettsia belli* provide a unique host-parasite-microbe system to empirically observe geographic patterns of parasite infestation and microbe infection that inform parasite ecology within the distribution of an invasive species. The objectives of our investigation were to (1) determine if core-periphery patterns of tick infestation rates occurred in cane toads. We predicted that there would be fewer tick infestations in toads at the periphery of the invasion than at the core of their distribution and (2) identify whether these invasive ticks were infected with exotic *Rickettsia* species not native to Florida and (3) determine if the pattern of a core-periphery distribution was maintained in the exotic bacteria found in ticks or cane toads. To conduct this study, we surveyed cane toad populations along a core to periphery distributional gradient for the presence of ticks. We then screened ticks and cane toads for infection with rickettsial bacteria.

## Materials and methods

### Cane toad and tick collection

We collected cane toads from 10 populations across their invasive range in Florida (Fig. 1) from April–June 2021. Sampling dates overlapped with the cane toad breeding season, which occurs from March through September in Florida (Krakauer 1968). We determined survey locations based on prior studies and citizen science reports through EDDMapS (Mittan & Zamudio 2019; Rubio et al. 2020; EDDMapS 2024). We conducted surveys near water bodies in urban areas at night, when and where cane toads actively forage (Wilson 2016). We captured toads by hand and placed them into individual ventilated plastic containers until ticks could be removed; animals were held in containers for < 24 h (University of Florida IACUC #202111387).

Toads were visually inspected for all life stages of attached ticks prior to euthanasia. If ticks were present, they were removed from the toad using fine tip forceps. Once removed, ticks were stored in 100% molecular-grade ethanol until they could be identified and extracted for DNA. We identified ticks to species morphologically using taxonomic keys from the United States, Central America, and South America (Keirans and Oliver 1993; Guzman-Cornejo et al. 2011; Nava et al. 2017).

After ticks were removed, toads were humanely euthanized through dermal application of 20% benzocaine gel (American Veterinary Medical Association 2020, IACUC #202111387). Following euthanasia, we collected morphological data (i.e., snout-urostyle length (SUL), mass, and sex) and tissue biopsies from all toads. When ticks were found on the toads, we took a skin biopsy at the site of tick attachment, referred to hereafter as attachment tissue. We collected vent tissue from all toads to assess if *Rickettsia* bacteria could be collected from highly vascularized tissues (Levin et al. 2016). Tissue samples were placed in 100% molecular grade ethanol and then stored at − 20 °C until they were extracted for DNA.

### DNA extractions

Tick and tissue samples were rinsed once with PBS buffer and twice with DI water to remove debris or benzocaine gel before DNA was extracted. DNA was then extracted from ticks and cane toad tissue samples using the Qiagen Gentra Puregene Kit (Valenica, CA, USA) with the manufacturer protocol. We extracted DNA from individual adults and nymphal ticks, but aggregated larvae into pools of 25 ticks per host. We cut vent and attachment site tissue into 10 ng pieces before extraction (Qiagen 2014). We stored eluted DNA at − 20 °C until PCR protocols were conducted.

### PCR and sequencing

To screen for *Rickettsia,* we initially targeted the gltA gene, as it can broadly detect *Rickettsia* species (Roux et al. 1997). We further analyzed *Rickettsia* positive samples using primers that amplified portions of two additional genes: atpA and coxA because they have previously been shown to successfully amplify *R. bellii* (Weinert et al. 2009; Krawczak et al. 2018). All three genes were used to differentiate between strains of *R. bellii*. We ran a positive and negative control for each PCR assay. We used a positive control from a *Rickettsia* sp. collected from an *Ixodes scapularis* specimen. Our negative control was PCR grade water. All PCR products were run on a 1.5% agarose gel with RedView Stain (Genecopoeia, Rockville, MD) and visualized on UVP gel documentation system (Analytik-Jena, Beverly, MA). We considered samples positive if they had the appropriate band size. All PCR products were cleaned with SAP/Exonuclease and sent to a commercial lab for Sanger sequencing (Functional Biosciences, WI, USA). Consensus sequences were constructed from forward and reverse primers for atpA and gltA. For coxA only forward sequences were used due to a bacterial coinfection of *Chryseobacterium sp*. that was co-amplified with the reverse primer (Supplementary Materials). Sequences were then aligned using Geneious software (2019.1.3) (Biomatters Ltd., Auckland, New Zealand) and compared to sequences in GenBank using NCBI BLAST.

### Phylogenetic and network analyses

We concatenated and assembled our three gene sequences in Geneious and compared them with sequence data from *R. bellii* isolates and field collected specimens deposited in GenBank (OP650115–OP650207). We aligned our samples with GenBank samples using ClustalW (Thompson et al. 1994) in MEGA X (Kumar et al. 2018) software. Each alignment was visually examined to make sure all sequences aligned.

To determine the relationship between our *Rickettsia* samples and other Rickettisial species, we constructed a tree using the Tamura-Nei genetic distance model and the UPGMA tree building method with 500 bootstrap. We then examined each target gene and all 3 genes concatenated in a minimum spanning network in PopArt (Leigh and Bryant 2015) in order to compare our samples from haplotypes in North America and South America. Additional sequences from Krawczak et al. (2018) and GenBank (Supplementary Table 1) supplemented the haplotype network.

## Results

### Cane toad and tick collections

We collected a total of 234 cane toads from 10 populations across south and central Florida. Surveys yielded 54 females and 180 males, including 212 adult toads and 22 juveniles. Snout-urostyle length ranged from 6.8 to 13.3 cm with an average of 10.8 ± 1.6 cm. Of all cane toads collected, 56/234 (23.9%) toads were infested with ticks (Table 1). Toads that were infested with ticks were collected from 3/10 populations: Homestead, Miami, and West Palm Beach (Fig. 1). Among the three infested toad populations, ticks were found on toads 60.0% (57/95 toads) of the time. All ticks were identified as *A. rotundatum*. In total we collected 495 *A. rotundatum* and toads were infested with 5.2 ± 1.5 ticks/toad (Table 1).

**Table 1:** Total number of ticks collected at each field site by life stage and average tick infestation per toad based on site. Tick infestation average is based only on cane toad populations with ticks. Standard error is reported for average tick infestation

Field site (N)	Larvae	Nymph	Adult	Total ticks collected	Average tick infestation
West Palm Beach (35)	41	24	9	74	2.3 (± 0.7)
Miami (21)	16	16	8	40	1.9 (± 0.7)
Homestead (39)	307	66	8	381	10.2 (± 3.2)
Total (75)	364	106	25	495	5.2 (± 1.5)

### Rickettsia screening

We tested ticks and tissue from vent and attachment sites for the presence of *Rickettsia* bacteria. Of the toads infested with ticks, 27/56 toads (48.2%) had ticks infected with *Rickettsia* (Table 2). Homestead, Miami, and West Palm Beach all had ticks with rickettsial infections. We did not detect *Rickettsia* in any vent or tick attachment tissue samples.

**Table 2:** Toads with ticks infected with *Rickettsia* bacteria based on gltA gene. Wilson Score 95% confidence interval was calculated for pathogen prevalence of *Rickettsia*

Field sites with ticks	Toads with *Rickettsia* positive ticks
West Palm Beach	7/15 (47%) (± 0.23)
Miami	1/9 (11%) (± 0.21)
Homestead	19/32 (59%) (± 0.16)
Total	27/56 (48%) (± 0.05)

### Phylogenetic and haplotype network analyses

**Fig. 2:**
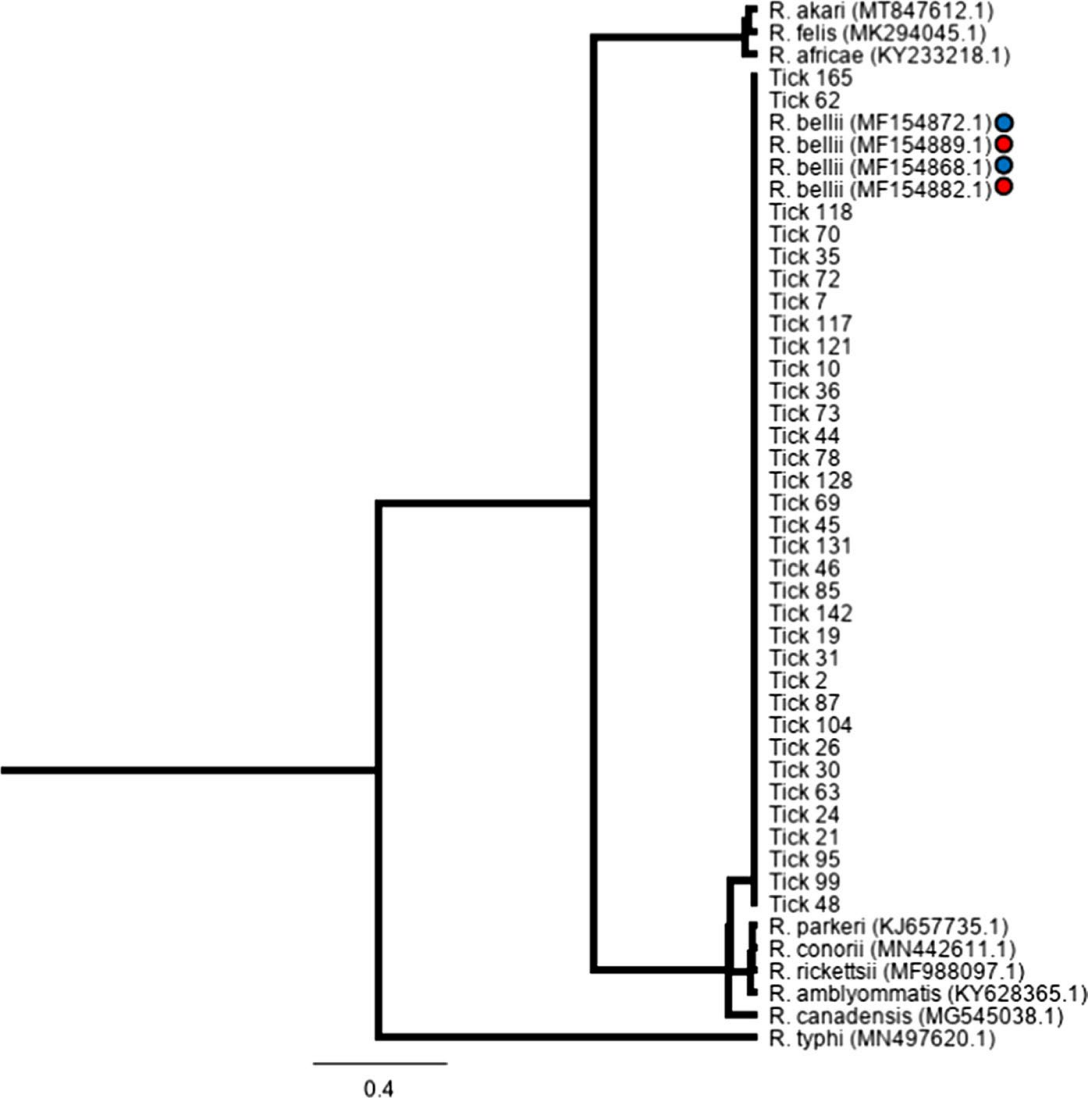
A phylogenetic tree for the gltA gene of *Rickettsia* species was constructed using the Tamura-Nei genetic distance model and the UPGMA tree building method with 500 bootstrap. Sequences from this study are designated as Tick #. Previously published sequences are listed by *Rickettsia* species and accession number. Blue dots indicate R. bellii collected from South America, and red dots are North America

Examining the gltA gene we found that our samples grouped with *R. bellii* and not with other rickettsial species (Fig. 2). We found that all three target genes from *A. rotundatum* also closely matched with *R. bellii* sequences from both North and South America (Table 3).

**Table 3:** Closest matches in GenBank to our *A. rotundatum* samples for Florida for each target gene

Pathogen species	Target gene	% Identical (Accession #)	Tick species	Loc locality
*Rickettsia bellii*	atpA	99.9%(MT009131)	*Amblyomma dissimile*	French Guiana
*Rickettsia bellii*	coxA	99.7%(CP000849)	*Dermacentor variabilis*	Ohio, USA
*Rickettsia bellii*	gltA	99.4% (MW384865)	*Amblyomma ovale*	El Salvador

**Fig. 3:**
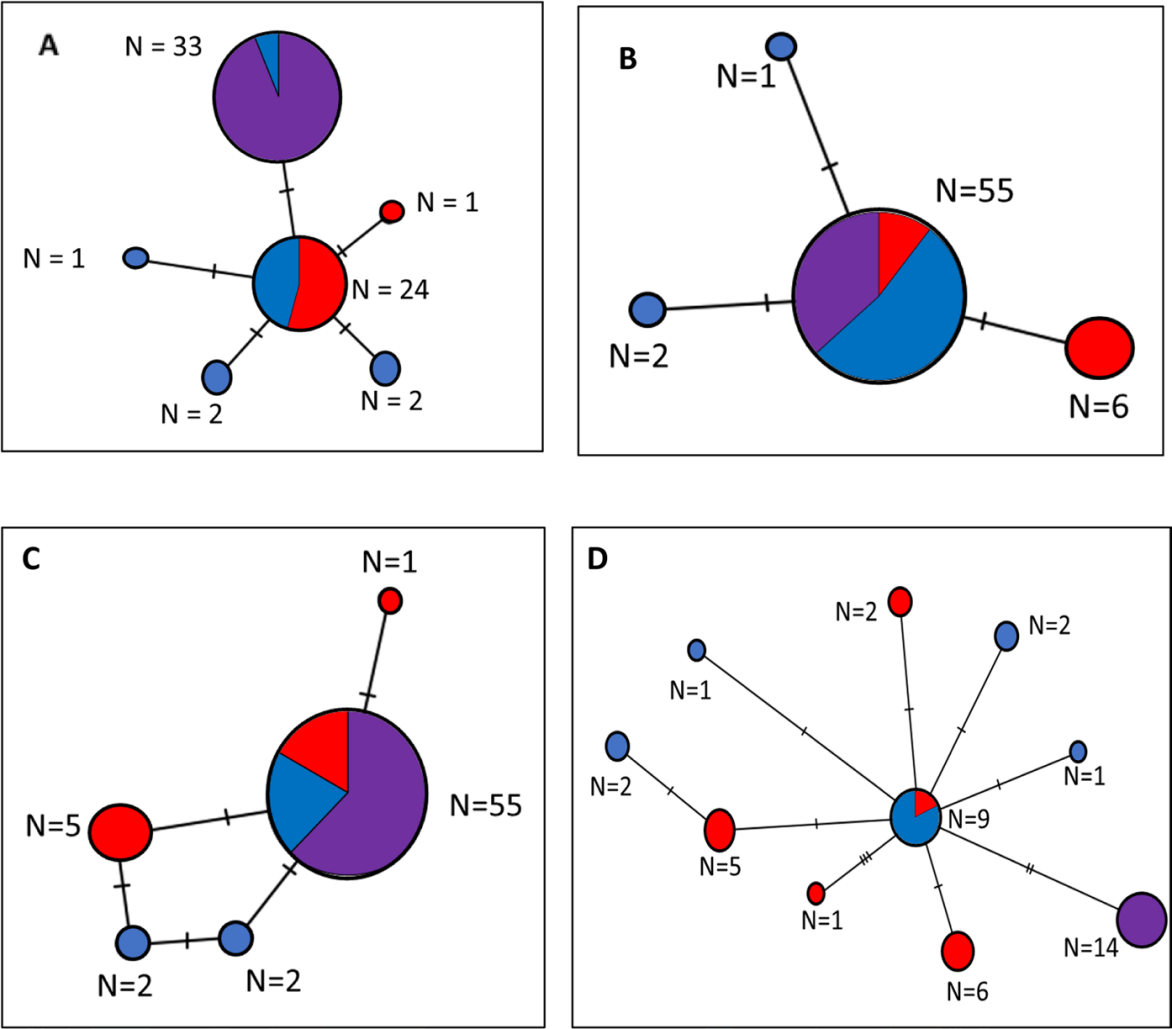
Haplotype networks for partial gene sequences of *Rickettsia bellii* and concatenated sequences. (A) atpA, (B) coxA, (C) gltA, (D) All genes concatenated. Red = North American R. *bellii*. Blue = South American *R. bellii*. Purple = Florida *R. bellii*

Using the minimum spanning network based on the atpA gene, we found that our samples shared the same haplotype as two samples from South America detected in *Amblyomma dissmile* (Fig. 3) (Supplementary Materials Table 1). However, there was not a clear distinction between North and South American haplotypes for any sequence or when all three sequences were concatenated (Fig. 3).

## Discussion

Our investigation aimed to determine whether tick infestation in cane toads and bacterial infection in ticks decreased from the core to the periphery of the cane toad’s distribution in Florida. We found evidence that cane toads at sites near the initial establishment were infested with *A. rotundatum*. Cane toads collected from Homestead, Miami, and West Palm Beach were infested with *A. rotundatum (*3/10 locations surveyed). These locations were along the south Atlantic coast of Florida where toad populations were introduced in the 1950’s and 1960’s (King and Krakauer 1966; Krakauer 1968; Wilson 2016). Interestingly, cane toads from Fort Lauderdale lay between two infested populations yet this population did not have ticks, which exemplifies the stochastic nature of ectoparasite infestation among host populations. None of the cane toad populations > 120 km from the core of the introduction contained toads that were infested with ticks. In relation to the core population, these uninfested, peripheral populations lay to the west on Gulf Coast side of Florida or to the north in West Palm Beach. Cane toad populations in these locations did not become established until the early 2000’s and were geographically distant from the founder populations (Wilson 2016). The lack of tick infestation along the invasion front is consistent with founder effects and provides support for the Enemy Release Hypothesis (Torchin et al. 2003). Invasive species can establish populations without parasites via stochastic processes or lose their parasites as they advance along the expansion front due to low host densities or unfavorable environmental conditions for the invasive tick species (Torchin et al. 2003; Phillips et al. 2010).

Unfavorable environmental conditions for parasites at the periphery of an expanding distribution can further facilitate founder effects, but we did not find evidence for this additive effect. We hypothesized that because cane toads in Florida have been shown to prefer disturbed and urbanized habitats that may hinder tick survival (Meshaka et al. 2006; Wilson 2016), it could limit the expansion of this tick species on cane toads. *Amblyomma rotundatum* is a 3-host tick, meaning it must drop from its host after feeding, then molt and wait for another bloodmeal. The environment into which ticks drop is crucial to their survival because ticks spend more than 99% of their lifetime off-host (Needham 1991, Diuk-Wasser et al. 2021). Cane toads are commonly found in manmade ponds, canals, and residential yards (Meshaka et al. 2006; Wilson 2016, pers. Obs.), and these urban habitats may not have the appropriate microclimate to prevent tick desiccation (Needham 1991). Urbanized habitats often lack preferred vegetation cover, moist soil, or leaf litter where ticks can take cover (Burtis et al. 2019; Diuk-Wasser et al. 2021). Studies in French Guiana (part of the native range of both cane toads and their ticks) have documented that urbanized habitat led to a loss of ticks on cane toads despite having sufficient toad populations to support tick transmission. In these cases, abiotic constraints in the urban environment prevented ticks from establishing (Devore et al. 2020). Nonetheless, we found ticks in the heavily urbanized core population of cane toads that have been established for more than 60 years, suggesting that local environmental conditions were not the driving factor limiting expansion on cane toad populations.

The core to periphery gradient in tick infestation of cane toads did not support the hypothesis that this distribution was driven by spillback of *A. rotundatum* to toads. The widespread distribution of this tick on multiple reptile hosts sympatric to peripheral cane toad populations provided opportunity for spillback in the northern and western populations of cane toads, yet these populations were uninfested. Because the pattern of infestation was structured along a core to periphery gradient, it was unlikely a result of spillback of ticks from other established or native species. Indeed, cane toads were likely the initial hosts and one of the dispersal vectors of *A. rotundatum* that provided the mechanism for spillover of this tick to multiple native and non-native reptile species throughout Florida (Oliver et al. 1993).

We found evidence of a likely exotic bacterial microbe throughout the tick populations that we sampled. We detected *R. bellii* in *A. rotundatum* at all three field sites where ticks were found. Thus, while tick dispersal among host toad populations may have been hindered, it appears that *Rickettsia* have successfully dispersed with their tick vector as predicted by their endosymbiont status and transovarial and transstadial transmission routes.

Previous investigations into the phylogenetics of *R. bellii* found that isolates collected from North America and South America formed separate clades and grouped together by tick genus and host preference (Krawczak et al. 2018). In North America, *R. bellii* has primarily been reported in *Dermacentor* species (Krawczak et al. 2018), while in South America it has been shown to infect ticks in the genera *Ixodes*, *Haemaphysalis* and *Amblyomma* (Krawczak et al. 2018). Even though North American and South America *Rickettsia bellii* form distinct clades, there are few polymorphisms that differentiate isolates (< 0.5%) (Krawczak et al 2018). We sequenced three rickettsial genes that each contained polymorphisms found in *R. bellii*. The atpA gene matched closely to *Rickettsia* sequences from *Amblyomma dissimile* in French Guiana*,* another avid reptile feeder in South America (Table 3). Vector-pathogen affiliation and haplotype data from this atpA gene suggest *R. bellii* originated from South America. However, the coxA and gltA genes resembled strains recovered from different tick species and locations (Table 3). This combination of gene sequence data could not clarify whether or not our *R. bellii* originated from South America. Thus, while the atpA gene is suggestive of a South American origin, further genomic analysis is needed to definitively determine the origin of *R. bellii* in Florida.

Studies of *Rickettsia* and toads thus far have only identified pathogens in ticks but not toad tissues (Luz et al. 2013; Horta et al. 2006). We did not detect *Rickettsia* species in attachment site or vent tissue collected from cane toads in our study. Tick attachment tissue examined in previous studies on cane toads in South America found that *A. rotundatum* bites can result in skin lesions (Luz et al. 2013). Whether or not these lesions can harbor tick-borne pathogens is still unknown. Future studies should collect tissue from lesions and attachment sites and test them for tick-borne pathogens.

## Conclusions

Overall, we found that *A. rotundatum* infests cane toads in areas < 120 km from the initial introduction sites in southern Florida, but not in populations further north or west. Cane toad populations in Homestead and Miami, sites at or near the initial introductions, continue to have tick infestations with *A. rotundatum.* The expansion of this tick species on cane toads into peripheral populations may have been restricted by demographic stochasticity associated with founder events rather than changes in habitat conditions from core to periphery. The core to periphery decline in infestation suggests that these processes are a more likely explanation than spill-back from established host populations because peripheral populations were sympatric with other reptiles hosting *A. rotundatum*. Our surveys found that *A. rotundatum* from cane toads were frequently infected with *Rickettsia bellii*, which was likely a South American strain that arrived in Florida with cane toads. This invasion system provides insight into how *Rickettsia* bacteria can be successfully transported during a host invasion and maintained during the expansion phase. Because many *Rickettsia* species are pathogenic, these findings suggest that pathogen pollution with exotic *Rickettsia* can impact human or animal welfare globally.

### Supplementary Information

Below is the link to the electronic supplementary material.

Supplementary file 1 (DOCX 16 kb)

## Data Availability

Sequence data with metadata have been deposited in NCBI Genbank.

## References

[CR1] Acevedo AA, Lampo M, Cipriani R (2016). The cane or marine toad, *Rhinella marina* (Anura, Bufonidae): two genetically and morphologically distinct species. Zootaxa.

[CR2] American veterinary medical association. (2020). AVMA guidelines for the euthanasia of animals: 2020.0.1 edition. 121 pp

[CR3] Barnett LK, Phillips BL, Heath ACG, Coates A, Hoskin CJ (2018). The impact of parasites during range expansion of an invasive gecko. Parasitology.

[CR4] Barré N, Uilenberg G (2010). Spread of parasites transported with their hosts: case study of two species of cattle tick. Rev Sci Tech.

[CR5] Burridge, MJ (2011) Non-native and invasive ticks. University Press of Florida

[CR6] Burtis JC, Yavitt JB, Fahey TJ, Ostfeld RS (2019). Ticks as soil-dwelling arthropods: an intersection between disease and soil ecology. J Med Entomol.

[CR7] Capinha C, Seebens H, Cassey P, García-Díaz P, Lenzner B, Mang T, Moser D, Pyšek P, Rödder D, Scalera R, Winter M, Dullinger S, Essl F (2017). Diversity, biogeography and the global flows of alien amphibians and reptiles. Divers Distrib.

[CR8] Chalkowski K, Lepczyk CA, Zohdy S (2018). Parasite ecology of invasive species: conceptual framework and new hypotheses. Trends Parasitol.

[CR9] Corn JL, Mertins JW, Hanson B, Snow S (2014). First reports of ectoparasites collected from wild-caught exotic reptiles in Florida. J Med Entomol.

[CR10] Cunningham AA, Daszak P, Rodriguez JP (2003). Pathogen pollution: defining a parasitological threat to biodiversity conservation. J Parasitol.

[CR11] DeVore JL, Shine R, Ducatez S (2020). Urbanization and translocation disrupt the relationship between host density and parasite abundance. J Anim Ecol.

[CR12] Diuk-Wasser MA, VanAcker MC, Fernandez MP (2021). Impact of land use changes and habitat fragmentation on the eco-epidemiology of tick-borne diseases. J Med Entomol.

[CR201] EDDMapS. 2024. Early Detection & Distribution Mapping System. The University of Georgia - Center for Invasive Species and Ecosystem Health

[CR13] El Karkouri K, Ghigo E, Raoult D, Fournier PE (2022). Genomic evolution and adaptation of arthropod-associated *Rickettsia*. Sci Rep.

[CR14] Ewen JG, Bensch S, Blackburn TM, Bonneaud C, Brown R, Cassey P, Clarke RH, Pérez-Tris J (2012). Establishment of exotic parasites: the origins and characteristics of an avian malaria community in an isolated island avifauna. Ecol Lett.

[CR15] Floerl O, Inglis GJ, Dey K, Smith A (2009). The importance of transport hubs in stepping-stone invasions. J Appl Ecol.

[CR16] Guglielmone AA, Nava S (2010). Hosts of Amblyomma dissimile Koch, 1844 and *Amblyomma rotundatum* Koch, 1844 (Acari: *Ixodidae*). Zootaxa.

[CR17] Guzman-Cornejo C, Robbins RG, Guglielmone AA, Montiel-Parra G, Pérez TM (2011). The *Amblyomma* (Acari: *Ixodida*: *Ixodidae*) of Mexico Identification keys, distribution and hosts. Zootaxa.

[CR18] Hanson BA, Frank PA, Mertins JW, Corn JL (2007). Tick paralysis of a snake caused by *Amblyomma rotundatum* (Acari: *Ixodidae*). J Med Entomol.

[CR19] Horta MC, Pinter A, Schumaker TTS, Labruna MB (2006). Natural infection, transovarial transmission, and transstadial survival of *Rickettsia bellii in* the tick *Ixodes loricatus* (Acari: *Ixodidae*) from Brazil. Ann N Y Acad Sci.

[CR20] De Jesus, C.E. 2021. Surveillance and ecology of tick-borne pathogens and tick-host associations of reptiles and amphibians in Florida. Dissertation Thesis. University of Florida

[CR21] Keirans JE, Oliver JH (1993). First description of the male and redescription of the immature stages of *Amblyomma rotundatum* (Acari: *Ixodidae*), a recently discovered tick in the U.S.A.. J Parasitol.

[CR22] King W, Krakauer T (1966). The exotic herpetofauna of Southeast Florida. Q J Fla Acad Sci.

[CR23] Krakauer T (1968). The ecology of the neotropical toad, *Bufo marinus*. South Fla Herpetol.

[CR24] Krawczak FS, Labruna MB, Hecht JA, Paddock CD, Karpathy SE (2018). Genotypic characterization of *Rickettsia bellii* reveals distinct lineages in the United States and South America. Biomed Res Int.

[CR25] Krysko KL, Burgess JP, Rochford MR, Gillette CR, CuevaD Enge KM, Somma LA, Stabile JL, Smith DC, Wasilewski JA, Others,  (2011). Verified non-indigenous amphibians and reptiles in Florida from 1863 through 2010: outlining the invasion process and identifying invasion pathways and stages. Zootaxa.

[CR26] Kumar S, Stecher G, Li M, Knyaz C, Tamura K (2018). MEGA X: molecular evolutionary genetics analysis across computing platforms. Mol Biol Evol.

[CR27] Labruna MB (2009). Ecology of *Rickettsia* in South America. Ann N Y Acad Sci.

[CR28] Labruna MB, Whitworth T, Bouyer DH, McBride J, Camargo LMA, Camargo EP, Popov V, Walker DH (2004). *Rickettsia bellii* and *Rickettsia amblyommii* in *Amblyomma* ticks from the state of Rondônia, Western Amazon. Brazil J Med Entomol.

[CR29] Laukaitis HJ, Macaluso KR (2021). Unpacking the intricacies of *Rickettsia*–vector interactions. Trends Parasitol.

[CR30] Leigh JW, Bryant D (2015). Popart: full-feature software for haplotype network construction. Methods Ecol Evol.

[CR31] Lever C (2001). *The cane toad: The history and ecology of a successful colonist*. Westbury Academic & Scientific Pub

[CR32] Levin ML, Snellgrove AN, Zemtsova GE (2016). Comparative value of blood and skin samples for diagnosis of spotted fever group rickettsial infection in model animals. Ticks Tick Borne Dis.

[CR33] Lillywhite HB, Sheehy CM, Lillywhite H, Martins M (2019). The unique insular population of cottonmouth snakes at seahorse key. Islands and snakes: isolation and adaptive evolution.

[CR34] Luong LT, Horn CJ, Brophy T (2017). Mitey costly: energetic costs of parasite avoidance and infection. Physiol Biochem Zool.

[CR35] Luz HR, Faccini JLH, Pires MS, da Silva HR, Barros-Battesti DM (2013). Life cycle and behavior of *Amblyomma rotundatum* (Acari: Ixodidae) under laboratory conditions and remarks on parasitism of toads in Brazil. Exp Appl Acarol.

[CR36] Luz HR, Silva-Santos E, Costa-Campos CE, Acosta I, Martins TF, Muñoz-Leal S, McIntosh D, Faccini JLH, Labruna MB (2018). Detection of *Rickettsia* spp. in ticks parasitizing toads (*Rhinella horribilis*) in the northern Brazilian Amazon. Experimental and Applied Acarology.

[CR37] Meshaka WE, DeVane J, Marshall SD (2006). An island of cane toads (*Bufo marinus*) in an ocean of xeric uplands in south-central Florida. Fla Sci.

[CR38] Mittan-Moreau CS, Kelehear C, Toledo LF, Bacon J, Guayasamin JM, Snyder A, Zamudio KR (2022). Cryptic lineages and standing genetic variation across independent cane toad introductions. Mol Ecol.

[CR100] Mittan CS, Zamudio KR (2019) Rapid adaptation to cold in the invasive cane toad *Rhinella marina*. Conservation Physiol 7(1):coy075. 10.1093/conphys/coy075 10.1093/conphys/coy075 PMC6379050 30800317

[CR39] Mooney HA, Cleland EE (2001). The evolutionary impact of invasive species. Proc Natl Acad Sci U S A.

[CR40] Nava S, Venzal JM, Acuña DG, Martins TF, Guglielmone AA (2017). Ticks of the Southern Cone of America: diagnosis, distribution, and hosts with taxonomy.

[CR41] Needham GR, Teel PD (1991). Off-host physiological ecology of ixodid ticks. Annu Rev Entomol.

[CR42] Oliver JH, Hayes MP, Keirans JE, Lavender DR (1993). Establishment of the foreign parthenogenetic tick *Amblyomma rotundatum* (Acari: *Ixodidae*) in Florida. J Parasitol.

[CR43] Parola P, Paddock CD, Socolovschi C, Labruna MB, Mediannikov O, Kernif T, Abdad MY, Stenos J, Bitam I, Fournier PE, Raoult D (2013). Update on tick-borne rickettsioses around the world: a geographic approach. Clin Microbiol Rev.

[CR45] Phillips BL, Brown GP, Shine R (2010). Life-history evolution in range-shifting populations. Ecology.

[CR46] Piotrowski M, Rymaszewska A (2020). Expansion of tick-borne rickettsioses in the world. Microorganisms.

[CR47] Polo G, Luz HR, Regolin AL, Martins TF, Winck GR, da Silva HR, Onofrio VC, Labruna MB, Faccini JLH (2021). Distribution modeling of *Amblyomma rotundatum* and *Amblyomma dissimile* in Brazil: estimates of environmental suitability. Parasitol Res.

[CR48] Prenter J, Macneil C, Dick JTA, Dunn AM (2004). Roles of parasites in animal invasions. Trends Ecol Evol.

[CR49] Qiagen. (2014). *Gentra® Puregene® Handbook*.

[CR50] Roux V, Rydkina E, Eremeeva M, Raoult D (1997). Citrate synthase gene comparison, a new tool for phylogenetic analysis, and its application for the rickettsiae. Int J Syst Bacteriol.

[CR200] Rubio AO, French CM Catenazzi A (2020) Morphological correlates of invasion in Florida cane toad (*Rhinella marina*) populations: Shortening of legs and reduction in leg asymmetry as populations become established. Acta oecologica 109:103652. 10.1016/j.actao.2020.103652

[CR51] Sakai AK, Allendorf FW, Holt JS, Lodge DM, Molofsky J, With KA, Baughman S, Cabin RJ, Cohen JE, Ellstrand NC, McCauley DE, O’Neil P, Parker IM, Thompson JN, Weller SG (2001). The population biology of invasive species. Annu Rev Ecol Syst.

[CR52] Sánchez-Montes S, Isaak-Delgado AB, Guzmán-Cornejo C, Rendón-Franco E, Muñoz-García CI, Bermúdez S, Morales-Diaz J, Cruz-Romero A, Romero-Salas D, Dzul-Rosado K, Lugo-Caballero C, Colunga-Salas P, Becker I (2019). *Rickettsia* species in ticks that parasitize amphibians and reptiles: novel report from Mexico and review of the worldwide record. Ticks Tick-Borne Dis.

[CR53] Thompson JD, Higgins DG, Gibson TJ (1994). CLUSTAL W: improving the sensitivity of progressive multiple sequence alignment through sequence weighting, position-specific gap penalties and weight matrix choice. Nucleic Acids Res.

[CR54] Torchin ME, Lafferty KD, Dobson AP, McKenzie VJ, Kuris AM (2003). Introduced species and their missing parasites. Nature.

[CR55] van Kleunen M, Dawson W, Maurel N (2015). Characteristics of successful alien plants. Mol Ecol.

[CR56] Weinert LA, Werren JH, Aebi A, Stone GN, Jiggins FM (2009). Evolution and diversity of *Rickettsia* bacteria. BMC Biol.

[CR57] Wilson, A. C. (2016). Distribution of cane toads (*Rhinella horribilis*) in Florida and their status in natural areas. PhD Thesis. University of Florida

