# Supplementary material for: Spatially limited pathogen pollution in an invasive tick and host system
Source: Biol Invasions. 2024 Apr 23;26(7):2037–47. doi: 10.1007/s10530-024-03291-9 (PMC11213802; doi:10.1007/s10530-024-03291-9)
Supplement: Supplementary file 1 — (DOCX 16 kb) [file 10530_2024_3291_MOESM1_ESM.docx]

We detected one other bacterium, a *Chryseobacterium* sp. from our tick samples. Our gltA primer was designed to target a 380 base pair portion of the citrate synthase gene in *Rickettsia*. *Chryseobacterium* also have a citrate synthase gene, which our primer set was able to amplify. Our primer set had 4 mismatches but was still able to bind the citrate synthase gene of *Chryseobacterium* and created a PCR product at 340 base pairs (Table S1).

**Table S1**. Primer binding sites of gltA Rickettsia primers to Chryseobacterium sp. (CP078104). Primer binding sites had a mismatch of 4, marked in red lettering. Primers bound to the citrate synthase gene of Chryseobacterium sp.

| Forward Primer | 5' GGG-GAC-CTG-CTC-ACG-GCG-G 3' |
| --- | --- |
| *Chryseobacterium* sp*.* | 5' GGG-GAC-CAC-TTC-ACG-GTG-G 3' |
|  |  |
| Reverse Primer | 5' ATT-GCA-AAA-AGT-ACA-GTG-AAC-A 3' |
| *Chryseobacterium* sp*.* | 5' AAT-GCA-AAC-ATT-ACG-GTG-AAC-A 3' |
